# Supplementary material for: Dual Effect of Organogermanium Compound THGP on RIG-I-Mediated Viral Sensing and Viral Replication during Influenza a Virus Infection
Source: Viruses. 2021 Aug 24;13(9):1674. doi: 10.3390/v13091674 (PMC8473027; doi:10.3390/v13091674)
Supplement: Supplementary file 1 [file viruses-13-01674-s001.zip › viruses-1322587-supplementary.pdf]

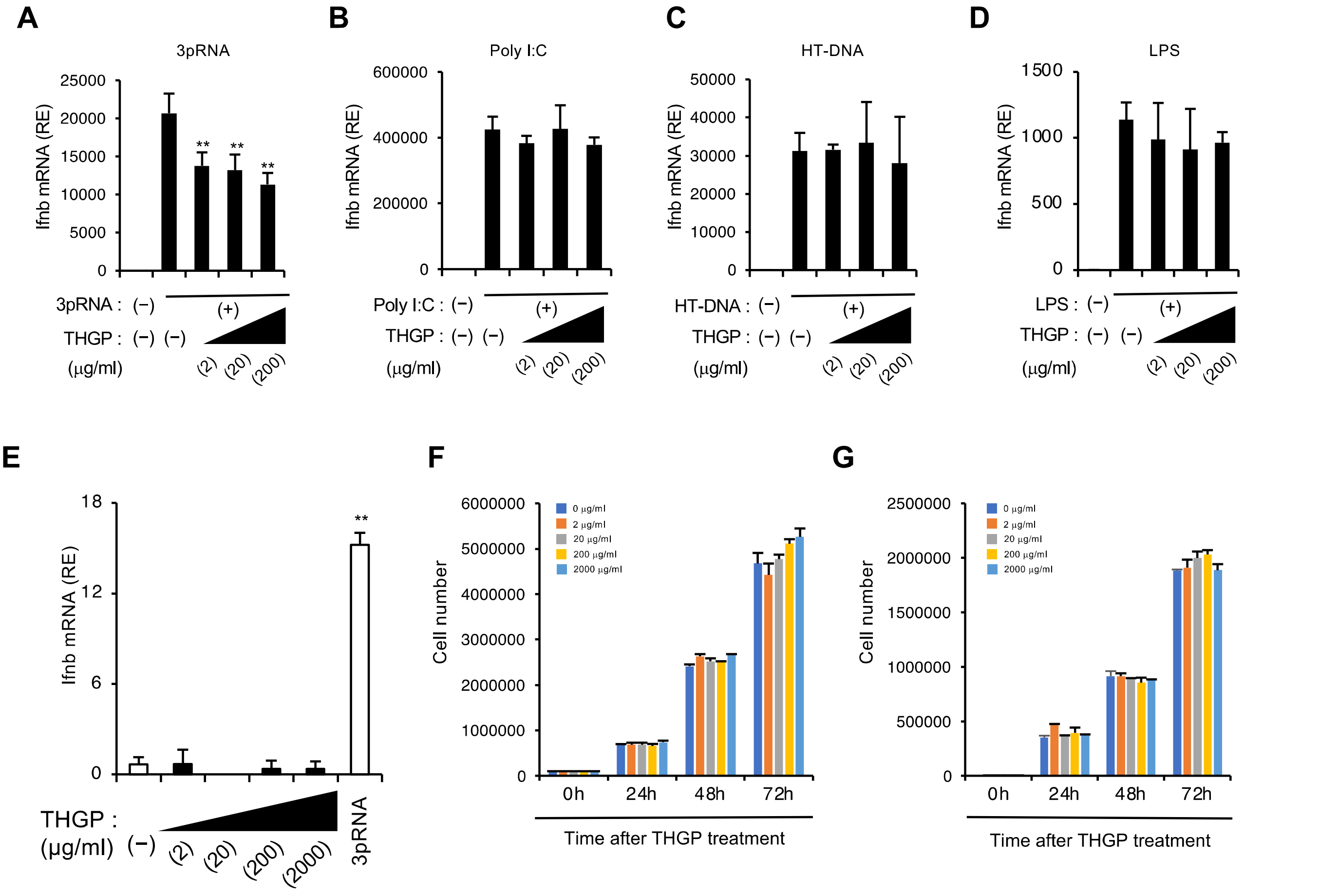

Figure S1. THGP suppresses IFN-β induction induced by 3pRNA but does not induce IFN-β itself and affect cell growth. **(A-D)** Quantitative RT-PCR (qRT-PCR) analysis of IFN-β mRNA levels at 8 h after stimulation with control or 3pRNA **(A)**, poly I:C **(B)**, HT-DNA **(C)**, and 2 h after stimulation with LPS **(D)** in RAW264.7 cells pretreated with indicated concentrations (0, 2, 20, 200, 2000 μg/ml) of THGP. **P < 0.01 vs control. **(E)** qRT-PCR analysis of IFN-β mRNA levels at 24 h after stimulation with indicated concentrations of THGP and 3pRNA as positive control in RAW 264.7 cells. (**F, G)** The cell number of RAW264.7 **(F)** and HEK293T **(G)** at indicated time after THGP treatment. Data are presented as mean and s.d. (n = 3) and are representative of at least three independent experiments.

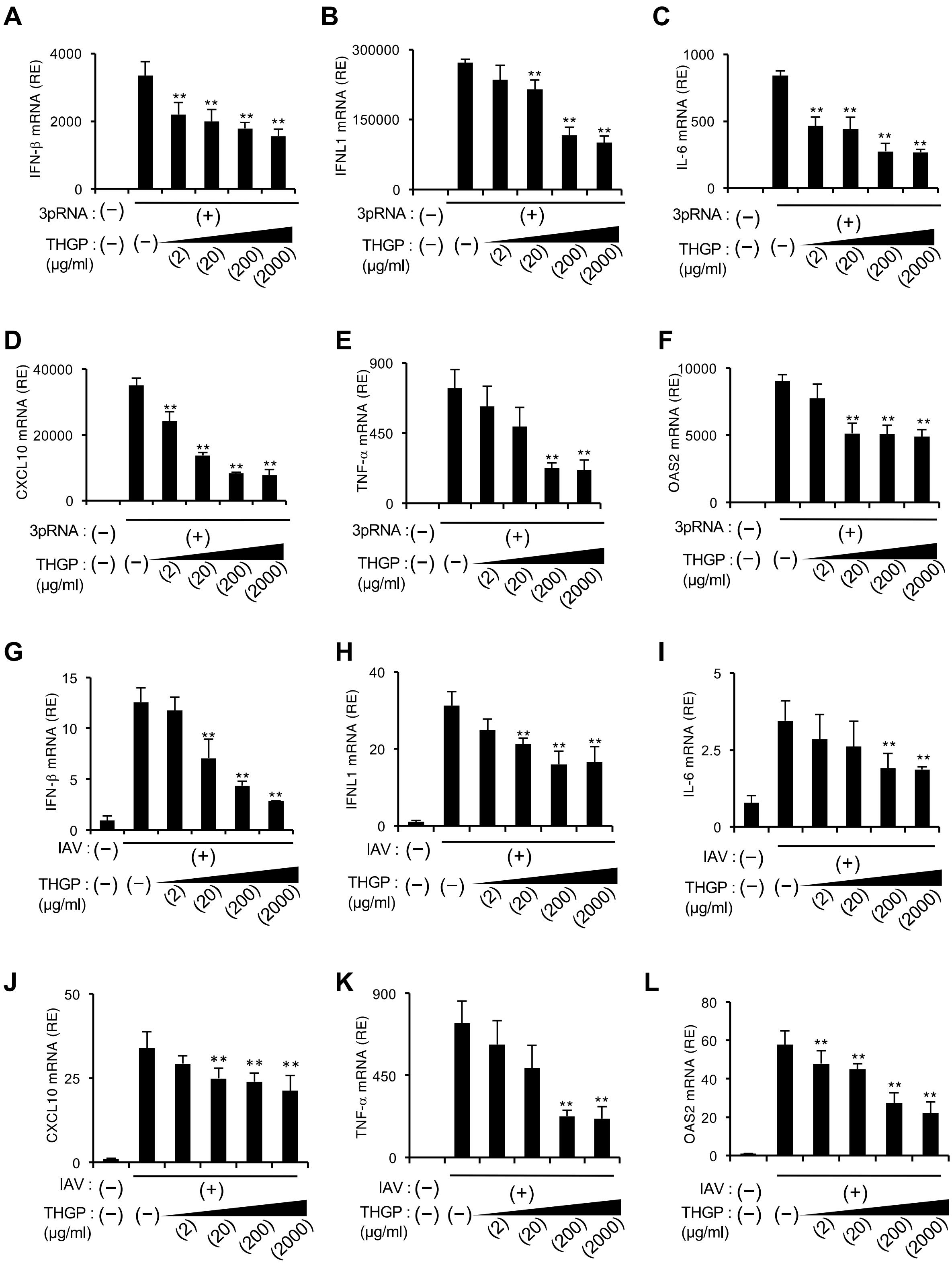

Figure S2. THGP reduces IFN induction in response to the stimulation with 3pRNA and infection with IAV in A549 cells. qRT-PCR analysis of IFN-β **(A, G)**, IFNL1 **(B, H)**, IL-6 **(C, I)**, CXCL10 **(D, J)**, TNF-α **(E, K)**, and OAS2 **(F, L)** mRNA levels at 8 h after stimulation with control or 3pRNA **(A-F)**, or infection with IAV **(G-L)** in A549 cells pretreated with indicated concentrations (0, 2, 20, 200, 2000 μg/ml) of THGP. **P < 0.01 vs control. NS, not significant.

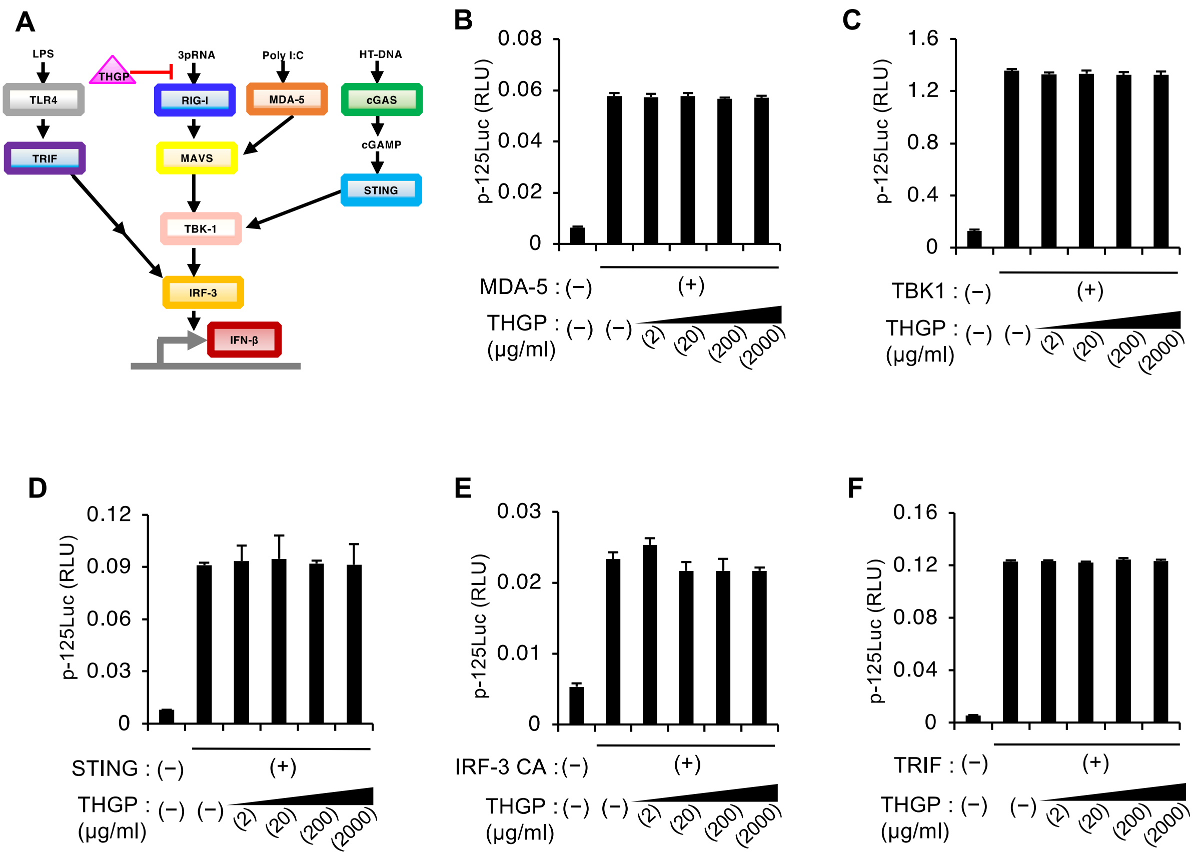

Figure S3. THGP does not affect *IFNB1* promoter activity upon overexpression of MDA-5, TBK-1, STING, IRF-3, and TRIF. **(A)** A scheme of the pattern recognition receptor-mediated pathways in this study and the position targeted by THGP are shown. **(B-F)** Luciferase assay of IFNB1 promoter after treatment of indicated concentrations of THGP following transfection of MDA-5 **(B)**, TBK1 **(C)**, STING **(D)**, constitutively active IRF-3 (IRF-3 CA) **(E)**, and TRIF **(F)** in HEK293T cells. Data are presented as mean and s.d. (n = 3) and are representative of at least three independent experiments.

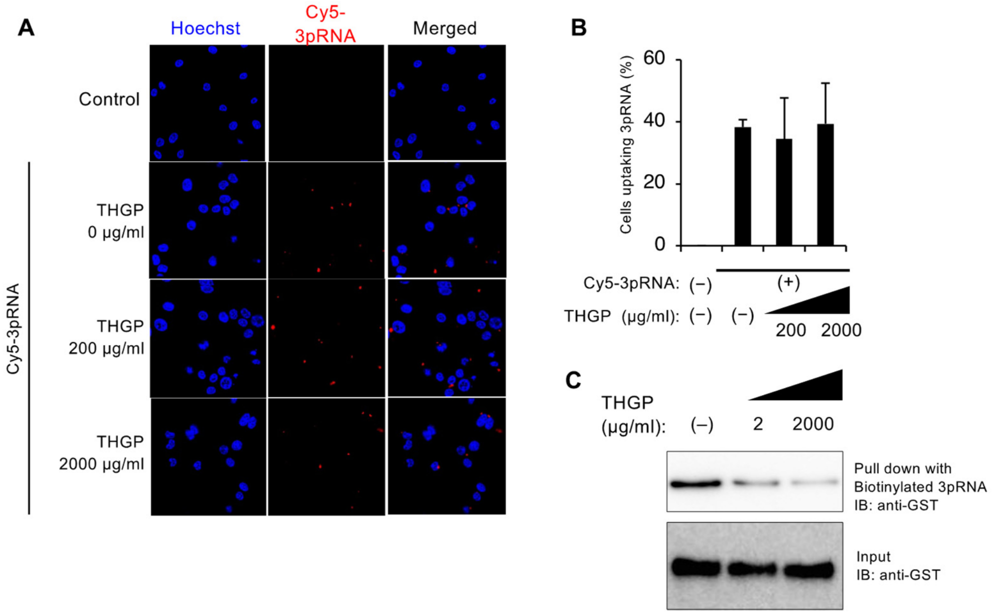

Figure S4. THGP does not influence the uptake of 3pRNA, but inhibits the interaction between 3pRNA and RIG-I. **(A)** RAW264.7 cells were transfected with Cy5-conjugated 3pRNA in the presence of indicated concentrations of THGP. After 2 h, cells were fixed and subjected to confocal microscopic analysis. **(B)** The quantification of **(A)** is shown. **(C)** 3pRNA

pull down assay, that test the interaction of biotinylated 3pRNA and GST-RIG-I recombinant proteins. Co-precipitated GST-RIG-I proteins with biotinylated 3pRNA in the presence of indicated concentrations of THGP were subjected to western blotting using anti-GST antibody.

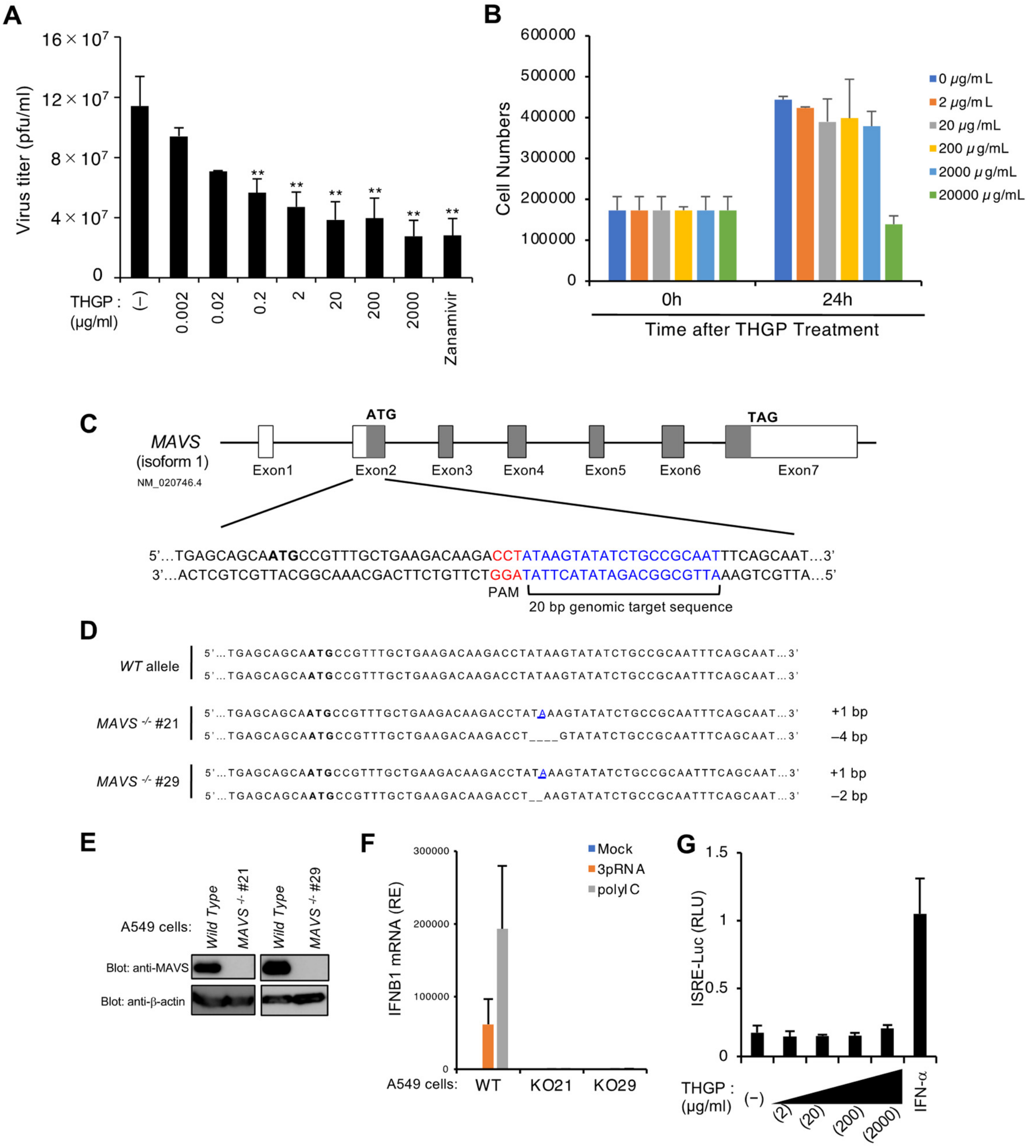

Figure S5. Effect of THGP on IAV replication in A549 cells, establishment of MAVS KO A549 cells and their IFN response to RNA ligands, and effect of THGP on ISRE response. **(A)** Viral titers measured in MDCK cells after 24 h of infection with IAV in A549 cells in the presence of the indicated concentrations of THGP or 30 μM Zanamivir as control. pfu, plaque-forming units. n = 3 samples per group. (B) The cell number of A549 cells at 24 h after the treatment with the indicated concentrations of THGP. The CC_{50} of THGP on A549 cells was 13,793.6 ± 2,291.3 μg/ml. Data are presented as mean and s.d. (n = 3) and are representative of at least three independent experiments. **(C)** Schematic depiction of the MAVS locus. The gray boxes indicate the MAVS open reading frame (ORF). The start codon (ATG) and stop codon (TAG) of ORF are shown in bold. The 20 bp genomic sequence targeted by the single guide RNA (sgRNA) is indicated by blue letters. The protospacer-adjacent motif (PAM) sequence is depicted in red. **(D)** Genomic DNA sequences of MAVS locus in the indicated wild-type (WT) and knock-out A549 cells. The sizes of the insertion (+) or deletion (–) are indicated to the right of each mutated allele. The inserted nucleotides are indicated in blue and underlined, and deleted nucleotides are indicated as underlined. The start codon (ATG) of ORF is shown in bold in the given sequence. All mutations cause frameshift and ablate MAVS protein expression. **(E)** Immunoblot analysis of MAVS protein and b-actin in A549 WT and MAVS KO cells.

(F) qRT-PCR analysis of IFNB1 mRNA levels at 8 h after stimulation with control, 3pRNA and poly I:C in A549 WT or MAVS KO cells. **(G)** Luciferase assay of ISRE promoter after treatment of indicated concentrations of THGP or 1,000 U of IFN-α in MAVS KO A549 cells.

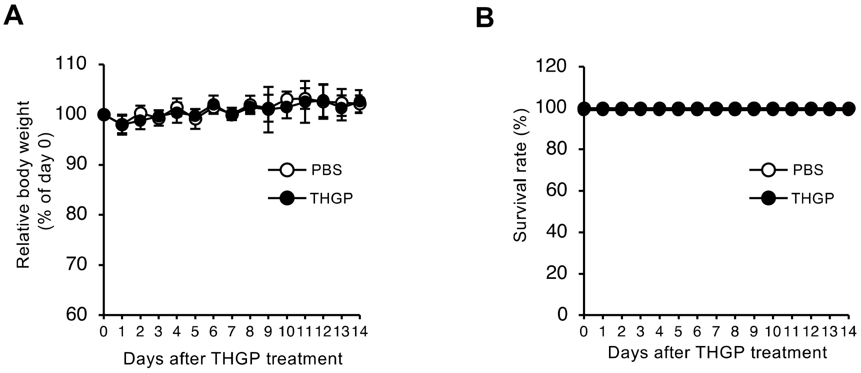

Figure S6. Effect of THGP on body weight and survival of uninfected WT mice. C57BL/6J mice were intranasally administrated with THGP or PBS at a dose of 50 mg/kg of body weight every 2 days for 14 days. Body weight change **(A)** and survival rate (**B**) were monitored (n=6 per group). Day 0 indicates the time of initiation of administration.

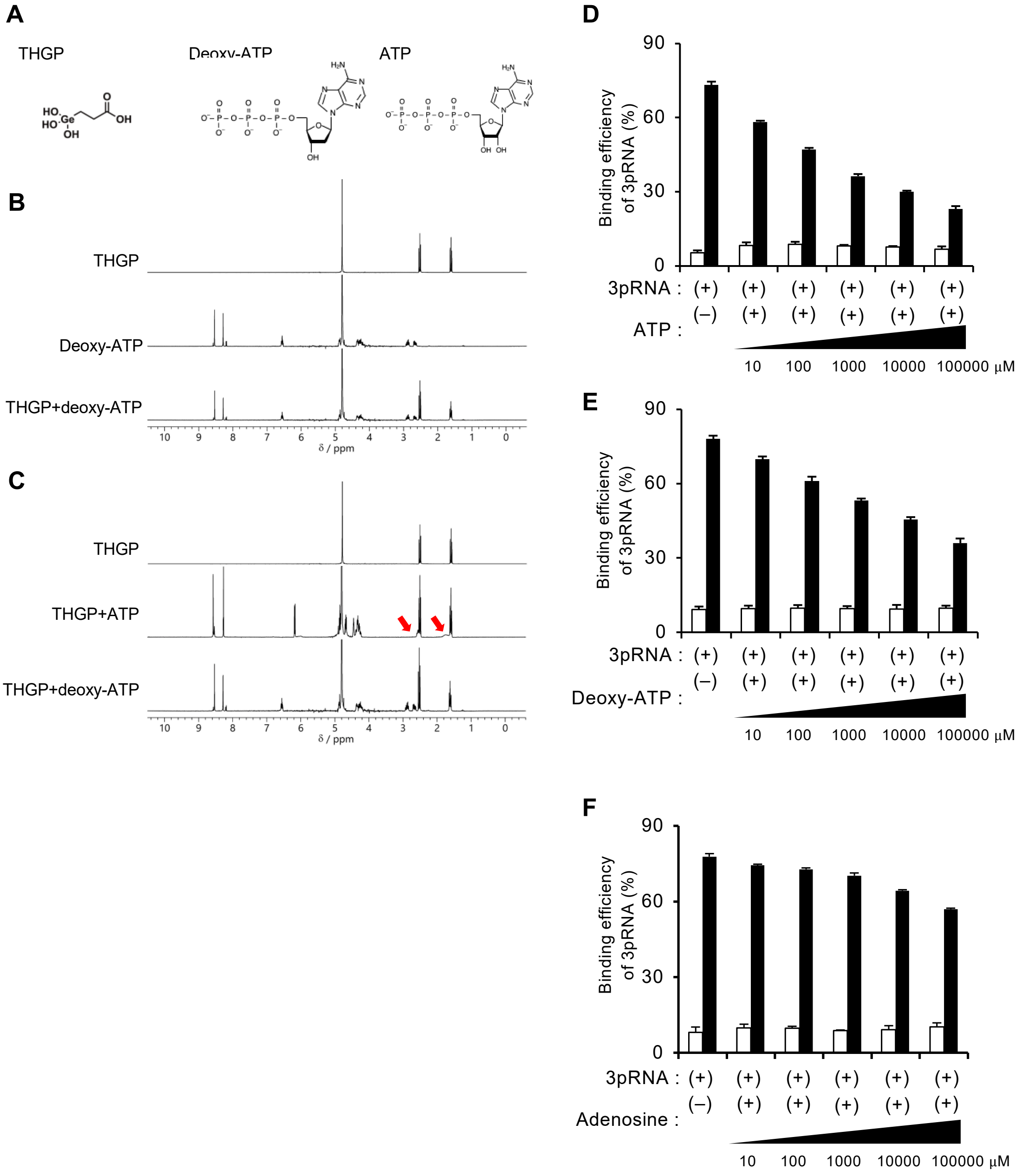

Figure S7. Supplemental information for the formation of a complex of 3pRNA and THGP. **(A)** Structure of THGP, deoxy-ATP, and ATP. **(B, C)** The formation of a complex between deoxy-ATP, ATP, and THGP was analyzed by ^{1}H-NMR (300 MHz). Red arrows indicated complex between ATP, and THGP. **(D-F)** THGP pull down assay of 3pRNA in the presence of ATP **(D)**, deoxy-ATP **(E)**, adenosine **(F)**. The % input of precipitated 3pRNA with THGP beads is shown. The IC_{50} values of ATP, deoxy-ATP, and adenosine are 912.01 μM, 2.37 mM, 27 M, respectively.

Table S1. gRNA Sequence of MAVS gene.

Gene	gRNA sequence
MAVS (human)	5′-ATTGCGGCAGATATACTTAT-3′

Table S2. Sequences of primers used for qPCR.

Gene target	Fw (5'→3')	Rv (5'→3')
IFNB1 (human)	ATGAC-CAACAAGTGTCTCCTCC	GCTCATGGAAAGAGCTG-TAGTG

Ifnb1 (mouse)	GAGCTCCAAGAAAGGAC-GAAC	GGCAGTGTAACTCTTCTGTAT
IL-6 (human)	AACCTGAACCTTCCAAA-GATGG	TCTGGCTTGTTCCTCACTACT
Il-6 (mouse)	TAGTCCTTCC-TACCCCAATTTCC	TTGGTCCTTAGCCACTCCTTC
TNFA (human)	ATGAGCACTGAAA-GCATGATCC	GAGGGCTGATTAGAGA-GAGGTC
Tnfa (mouse)	CCCTCACAC-TCAGATCATCTTCT	GCTACGACGTGGGCTACAG
IFNL1 (human)	CGCCTTGGAAGAGTCACTCA	GAAGCCTCAGGTCCCAATTC
CXCL10 (human)	GTGGCATTCAAGGAGTACCTC	GCCTTCGATTCTGGATTCAG-ACA
OAS2 (human)	AACTGCTTCCGACAATCAAC	CCTCCTTCTCCCTCCAAAA
GAPDH (human)	CATGA-GAAGTATGACAACAGCCT	AGTCCTTCCACGATACCAAAGT
Gapdh (mouse)	AGGTCGGTGTGAACGGATTTG	TGTAGACCATGTAGTT-GAGGTCA
Firefly luciferase	GTGGTGTGCAGCGAGAATAG	CGCTCGTTGTAGATGTCGTTAG
IAV PR8 NP RNA	GATTGGTGGAATTGGACGAT	AGAGCACCATTCTCTCTATT
